# High protein flexibility and reduced hydration water dynamics are key pressure adaptive strategies in prokaryotes

**DOI:** 10.1038/srep32816

**Published:** 2016-09-06

**Authors:** N. Martinez, G. Michoud, A. Cario, J. Ollivier, B. Franzetti, M. Jebbar, P. Oger, J. Peters

**Affiliations:** 1 Institut Laue Langevin, F-38042 Grenoble Cedex 9, France; 2 Univ. Grenoble Alpes, IBS, F-38041 Grenoble Cedex 9, France; 3 Univ. Brest, CNRS, Ifremer, LM2E, IUEM, 29280, Plouzané, France; 4 Univ Lyon, ENS Lyon, CNRS UMR 5276, F - 69364 Lyon cedex 07, France; 5 Univ Lyon, INSA Lyon, CNRS UMR 5240, F - 69621 Villeurbanne cedex, France; 6 Univ. Grenoble Alpes, LiPhy, F-38041 Grenoble Cedex 9, France

## Abstract

Water and protein dynamics on a nanometer scale were measured by quasi-elastic neutron scattering in the piezophile archaeon *Thermococcus barophilus* and the closely related pressure-sensitive *Thermococcus kodakarensis*, at 0.1 and 40 MPa. We show that cells of the pressure sensitive organism exhibit higher intrinsic stability. Both the hydration water dynamics and the fast protein and lipid dynamics are reduced under pressure. In contrast, the proteome of *T. barophilus* is more pressure sensitive than that of *T. kodakarensis*. The diffusion coefficient of hydration water is reduced, while the fast protein and lipid dynamics are slightly enhanced with increasing pressure. These findings show that the coupling between hydration water and cellular constituents might not be simply a master-slave relationship. We propose that the high flexibility of the *T. barophilus* proteome associated with reduced hydration water may be the keys to the molecular adaptation of the cells to high hydrostatic pressure.

The majority of Earth’s biotopes is represented by oceans below 1000 meter depth, sub-seafloor and deep-subsurface continental settings, referred to as the deep biosphere^1^,^2^. Hence, most biotopes on Earth experience high hydrostatic pressures (HHP). The most intriguing biotopes of the deep-biosphere are deep-sea hydrothermal vents, where organisms experience concomitantly high hydrostatic pressure and steep gradients in temperature, salinity or redox potential^3^. The deep-biosphere is populated by HHP-adapted organisms (piezophiles), whose diversity has been shown to extend to all three domains of life (*Eukarya*, *Bacteria* and *Archaea*)^2^,^4^. Physically the impact of pressure bears resemblance to both a lowering of temperature, since it will reinforce the structure of some molecules, such as membrane lipids, and reduce the molecular dynamics, and an increase in temperature, since it will as well destabilize other structures, such as proteins. In response to HHP stress, piezophiles have been shown to adapt by changing the lipid composition of their membranes or by producing small organic osmolytes^2^, but very little is known about synergistic structural and dynamical adaptation of macromolecules. In broad strokes and according to Le Chatelier’s principle, compression by HHP will favor a volume reduction and thus lead to reduced dynamics. Pressure impacts both structure and activity of biological macromolecules and features (proteins, membranes, cells, etc.) with specific effects. Indeed, increased pressure leads to higher membrane-lipid packing and reduced membrane functionality due to restricted lipid motion. In proteins, the reduction of the volume under pressure will impact protein activity, leading to a decrease of internal cavities although they stay dry. Upon higher pressures application, water intrusion will change the molecular bounds and structure and lead to a cold denaturation of the system^5^,^6^. In contrast to the adaptation to other stresses such as salinity or temperature for which there is a clear evidence for the structural adaptation of proteins, we still do not know what drives the difference at the molecular level in pressure tolerance between surface-adapted, piezosensitive organisms, which cannot grow at high pressure, and deep-sea adapted, piezophilic organisms, which require high hydrostatic pressure for growth.

The study of molecular dynamics in whole cells *in vivo* is extremely difficult as cells are highly complex systems. Consequently, only few of them were studied so far by means of incoherent neutron scattering, among them *Escherichia coli*^7^,^8^ (*E. coli*), *Haloarcula marismortui*^9^, red blood cells^10^, *Halobacterium salinarum*^11^,^12^ and very recently *Shevanella Oneidensis*^13^. The advantages of neutron scattering consist in the huge incoherent cross section of hydrogen with respect to other atoms present in biological samples, which allows studying cellular constituents^8^ as well as water dynamics^14^, and in the fact that unlike X-rays, thermal neutrons have such a low energy that they do not induce radiation damage to the sample. We are interested in both water and cellular dynamics, and how they respond to HHP, in order to understand the molecular mechanisms underlying adaptation to HHP. The cells were investigated in D_2_O at 293 K, although they are in a metabolically inactive state, but their membranes are more compact and impermeable at this temperature suppressing by the way H_2_O/D_2_O exchange. The fact that we can identify water motions in the QENS data although we are using D_2_O is due to the fact that significant quantity of H_2_O remains inside the cells and even in the interstitial volume between the cells. So even in case of some exchange, our data still allows to calculate proper parameters for each populations of water, as the incoherent scattering cross-section of hydrogen is 40 times that of deuterium^15^.

It has been shown by multiple techniques such as NMR^16^, small angle scattering^17^ and QENS^18^ that proteins and membranes^19^ have a hydration layer that differs significantly from the bulk phase. The presence of the hydration layer is capital for biological function^20^, as it allows the protein to perform a dynamical transition^21^, a mandatory condition for being functional. Depending on the used technique and the system under investigation the hydration water was sometimes found considerably slowed down with respect to the bulk phase^22^,^23^,^24^, and sometimes resembles to the characteristics of bulk water^13^,^25^,^26^,^27^,^28^ (cf. Fig. 1). One should notice that the exact value of the diffusion coefficient depends strongly on the time and space window at which it is obtained, so that differences extracted from NMR or QENS results are not surprising. Molecular dynamics simulations illustrate this point for lipid dynamics, where the diffusion coefficient in several motional regimes is very different^29^. Also, we would like to highlight that depending on the system under consideration, the hydration dynamics can greatly differ. For example, it has been shown that hydration water rotational correlation times differ for different proteins^30^, and this latter parameter is also affected by the state of the protein (native, denatured, molten globule or fibrillated)^30^,^31^. The origin of these discrepancies should be investigated systematically, as more as the exact nature of the protein-water interactions is still under debate^32^. Nevertheless, the study baring the most resemblance to our work^33^ in terms of time/space window and system under consideration has demonstrated that hydration water in *E. coli* is slowed down by a factor 15 with respect to the bulk, which is in the same order of magnitude of our findings. Water molecules are known to perform translational and rotational motions simultaneously, whereas we cannot specify more precisely the diffusive motions present in biomolecules (cf. Fig. 1). QENS has been successfully used to investigate the dynamical properties of proteins^21^, membranes^34^ and water^35^ of *in vivo* samples under several external conditions. QENS appears therefore as the method of choice to investigate whether the dynamics of the cell differs between HHP-adapted and HHP-sensitive microorganisms.

To address this question, we have investigated the molecular dynamics of two closely related hyperthermophilic archaeal species from the *Thermococcales* order. The piezophilic archaeon *Thermococcus barophilus* was isolated from the Snake Pit hydrothermal vent site of the mid Atlantic ridge at a depth of 3550 m^36^. The piezosensitive archaeon *Thermococcus kodakarensis* was isolated from a surface solfatara of Kodakara island, Japan^37^. Beside their respective HHP tolerance, these two species share similar growth requirements; they grow optimally at 85 °C and are genetically closely related, making it the ideal microbial system to monitor pressure adaptation mechanisms. The dynamics of whole cells of these two species were monitored by QENS on the spectrometers IN5^38^, IN6^39^ and IN16B^40^ at the Institut Laue-Langevin at 0.1 MPa and 40 MPa, which represent the optimal pressure conditions for *T. kodakarensis* and *T. barophilus*, respectively. We show here that the *T. barophilus* cells exhibit contrasted pressure-dependence with *T. kodakarensis* cells, including less hydration water showing significantly reduced dynamics under pressure and a pressure-sensitive proteome. These structural differences might be keys to the adaptation to HHP in *T. barophilus*.

## Results

### QENS data analysis

**Figure 2:**
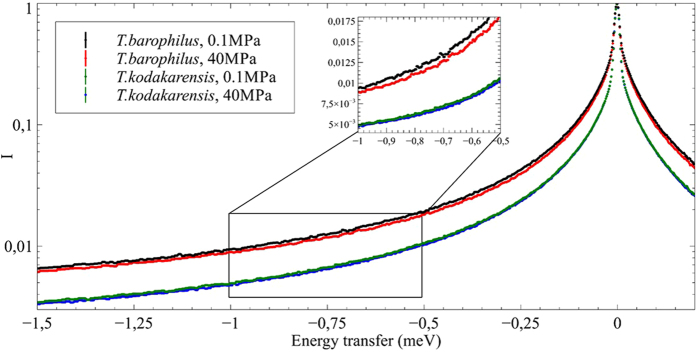
Normalized spectra averaged over all scattering angles for both samples at ambient pressure and at 40 MPa. The insert is a magnification of the energy transfer domain between −1. and −0.5 meV highlighting the differences of the curves observed between *T. barophilus* and *T. kodakarensis* samples, and between *T. barophilus* samples under 0.1 and 40 MPa.

QENS data presented in the Results section were acquired on the IN5 time-of-flight spectrometer at 10 μeV energy resolution on whole cells of *T. kodakarensis* and *T. barophilus* pellets, at two pressure points, e.g. ambient hydrostatic pressure (0.1 MPa) and 40 MPa (Fig. 2). The two chosen pressures have been shown in a previous study on IN13 to correspond to two different molecular dynamic regimes in *T. barophilus* cells^41^. To verify the integrity of the cells after the neutron experiment, an additional spectrum was recorded after pressure release. The spectra acquired prior and after the experiment were identical over the whole Q range (see details in Electronic Supplementary Information, ESI), showing that the integrity of the cells had not been affected during data acquisition. The approach to analyze QENS data is introduced in detail in the Methods section. As a general rule, a QENS spectrum of immobile particles would be a simple delta peak, whereas a perfect liquid would result in a single broad Lorentzian. Here we note that the *T. kodakarensis* curve is narrower than the *T. barophilus* one, thus indicating reduced mobility, e.g. stronger intramolecular non-covalent bounds can be assumed for this system and therefore a higher stability. Stability is defined as the force constant or resilience which can be extracted from elastic incoherent neutron scattering data as function of temperature^42^. To date, there are no defined procedures to determine it as a function of pressure. However, a hint at stability can be extracted from QENS data through the width of the different curves, as it stands for more or less motion.

The same conclusions are supported by data acquired on the spectrometer IN6 at ILL with an energy resolution of 100 μeV (ESI, Figure S2). Small but significant variations are observed as a function of pressure in *T. barophilus* demonstrating a reduction of the molecular dynamics as a function of pressure, as expected according to Le Chatelier’s principle.

### Contribution of different dynamical populations to QENS data

**Figure 3:**
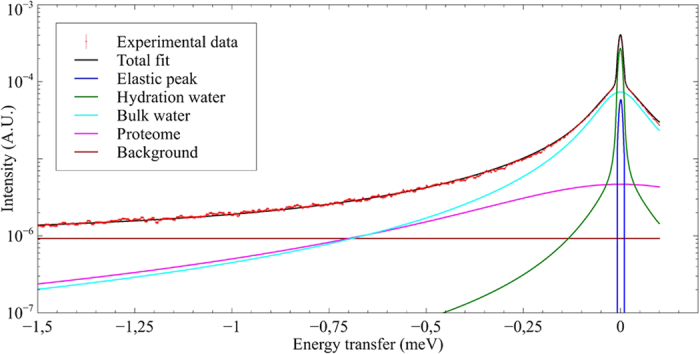
Experimental data and fit for *T. barophilus* at ambient pressure and Q = 0.75 Å^−1^.

Based on the concept of Brownian motion in a fluid it is possible to establish a theory describing diffusion in such a medium. Each singular diffusional motion can be described by a Lorentzian curve, hence the QENS signal can be fitted by a sum of Lorentzian functions (see equation (1) in Methods). In practice, the data do not permit to distinguish more than three or four contributions. However, these individual curves characterize specific motions inside the samples and allow extracting typical parameters which depict the motional behavior of one given class of particles. In the case studied here, the spectra as function of the modulus of the momentum transferred between neutron and sample, *Q*, could be fitted with a model describing the dynamics of four contributors (Fig. 3 and Data analysis section): The first component was associated with freely moving water, e.g. bulk water; the second component corresponds to the dynamics of biological macromolecules, represented mostly by the proteome; the third component corresponds to a second population of water molecules with slower motion due to confinement, e.g. hydration water. The last contribution is the elastic peak, which contains the signal of particles seen as immobile. Among the fit parameters which can be determined in such a way are diffusion coefficients D, which specify the (rotational or translational) transport of particles due to a random motion within a limited or an unlimited space and a residence time τ characterizing the typical time a particle performs oscillatory motions before diffusing again continuously.

### Different water populations characterize piezophilic and piezosensitive cells

The surfaces under the elastic peak and the various Lorentz curves correspond to the proportions of each of the populations contributing to neutron scattering. p_elastic_, p_hyd_, p_bulk_ and p_proteome_ represent the immobile particles, the hydration shell, the bulk water and the proteome, respectively. These factors are not affected by variations of pressure (Table 1), which implies that pressure does not change the water/proteome mass ratio. The part of protons included in the biomolecules is very similar for both samples (~0.23 ± 0.03). The proportions of water and biomolecules correspond well to what is reported for bacterial cell pellets, with around 70–80% of total water content^43^. However, the proportion of bulk water hydrogen atoms is smaller in *T. kodakarensis*, e.g. 0.60 vs 0.67 in *T. barophilus*, and the contribution of hydration water molecules is enhanced from 0.10 in *T. barophilus* to 0.17 in *T. kodakarensis*. These proportions are very similar to values reported for other cells in the literature^10^, which permits to safely attribute these components to bulk and hydration water. p_elastic_ is slightly increased from 0.01 in *T. barophilus* to 0.03 in *T. kodakarensis*. The latter indicates a higher proportion of immobile particles. Together with the increased number of water molecules bound at the surface, these findings substantiate the fact that *T. kodakarensis* appears as more stable in terms of intramolecular non-covalent and hydrophobic bounds (Fig. 2).

**Figure 4:**
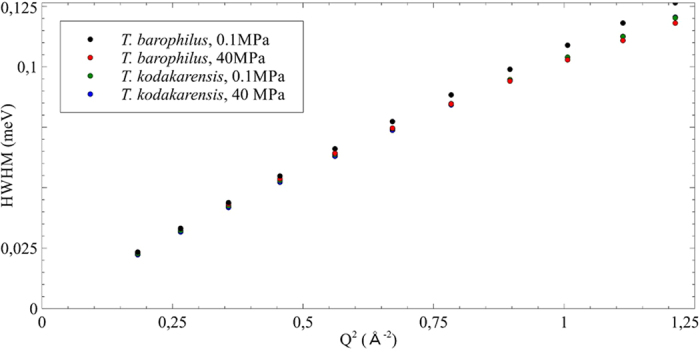
HWHM of the translational bulk water component for both samples at different pressure values as a function of Q^2^.

The bulk water component was fitted with a jump diffusion model convolved with a free rotation^44^. Indeed, the water molecules are restricted in space giving rise to a motion which deviates from free diffusion at large Q-values above 0.8 Å^−1^. In the model, water molecules perform a motion where they diffuse for a short time with a diffusion coefficient D_T_ and then vibrate at their position for a certain time τ, after which they perform another jump, while executing a free rotational motion with a rotational diffusion coefficient D_R_ (Table 1). The corresponding half width half maximum (HWHM) of the Lorentz function of the translational component for the two samples is shown in Fig. 4. The rotational component was also analyzed, but is not shown here, as it is independent of Q.

The width of a Lorentzian distribution describing unlimited diffusion, e.g. Brownian motion, as function of the momentum transfer squared would present a linear behavior, with D_T_ being the slope of the curve^44^. When water is limited in its motions by any kind of confinement, the HWHM presents a deviation from the purely linear behavior, which can be characterized by the two parameters D_T_ and τ, D_T_ corresponding now to the slope of the curve at low Q and τ being the inverse of Γ_∞,_ the constant value to which tends the HWHM at high Q. Here in the low Q regime, all four curves are extremely similar, so not surprisingly the translational diffusion coefficient is the same for all samples, regardless of the pressure. These observations are confirmed by other data obtained on IN6 at ILL, which is mostly sensitive to proteome and water motions (ESI, Figure S2). Moreover, the D_Tbulk_ value, e.g. 1.98 10^−5^ cm^2^/s (Table 1), was found to be close to that of pure bulk water, which is 2.3 10^−5^ cm^2^/s at 298 K^44^. In contrast, the residence time τ, which is related to interactions between molecules, is expected to be enhanced due to exchange mechanisms of biomolecules with hydration water^14^ and indeed this parameter seems much more affected in real systems^7^,^9^,^10^. In the present study, we observe a slight increase of the residence time from 1.05 to 1.55 in *T. barophilus* under HHP. The higher value could support a stronger confinement effect under HHP as this system is more sensitive to pressure. In contrast, both calculated values for *T. kodakarensis* are close to that of pure water. Unfortunately, due to the limited Q-range accessible by the spectrometer at this particular energy resolution, this trend is not statistically supported.

### Hydration water is more confined under pressure in *T. barophilus*

**Figure 5:**
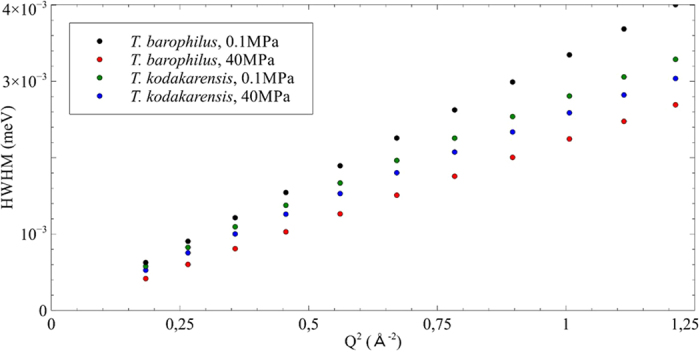
HWHM of the translational hydration water component for both samples at different pressures as a function of Q^2^.

The hydration water was fitted with a jump diffusion model convolved with a free rotation. The HWHM of the translational component is shown in Fig. 5. Notice that the energy scale of the HWHM differs by about a factor of 25 between the two water populations (Figs 4 and 5), indicating that the bulk water motions correspond to much shorter times than the hydration water ones. Such findings and the order of magnitude are in good agreement with values reported for the HWHM of water populations found in red blood cells^10^. However, the differences between the two species and for two pressure values are significant. One can see that while in *T. kodakarensis* pressure has almost no effect apart from a slight reduction in the diffusion coefficient (Table 1), the increase of hydrostatic pressure significantly reduces the quasi-elastic broadening of the hydration water component in *T. barophilus*. The impact of hydrostatic pressure on the hydration water and proteome dynamics was also studied up to 120 MPa on the spectrometer IN16B at the ILL (ESI, Figure S3). Intensity summed over all scattering angles in the elastic peak region as a function of pressure clearly shows that there is no increase for *T. kodakarensis* to at least 120 MPa, while there is a significant increase of the intensities for *T. barophilus* by 1.45 times between 0.1 and 120 MPa. This confirms the high stability of *T. kodakarensis* against pressure, and the reduction of the flexibility for *T. barophilus* as a function of pressure. The calculated values for D_Thyd_ are about an order of magnitude slower than in red blood cells^10^, but they are close to values found in neural tissue^45^ and recently for hydration water in protein powders^24^. The residence time, which is normally extracted from the high Q plateau, could not be calculated with our data due to the limited Q-range of the instrument at this particular resolution.

### The *T. barophilus* proteome is more flexible and flexibility increases with pressure

Proteins are the major constituents in microorganisms^46^, thus their dynamics dominates the component associated with biomolecules. DNA represents less than 3% of the dry bacterial mass^47^, we thus can safely neglect its contribution. The large Lorentzian component, characterized by its HWHM Γ_proteome_, has the most surprising behavior. Indeed, the two systems have rather different Γ_proteome_ values, confirming again the higher stability of *T. kodakarensis* with respect to *T. barophilus*. Furthermore, Γ_proteome_ for *T. kodakarensis* undergoes a slight reduction from 0.363 meV to 0.347 meV at 40 MPa indicating a reduction in dynamics, whereas for *T. barophilus* it increases with increasing pressure, indicating an enhancement in dynamics (Table 1). Similar results were recently obtained from QENS measurements on an oligomeric protein from deep sea hyperthermophile (Inorganic pyrophosphatase (IPPase) from *Thermococcus thioreducens*) in comparison with the mesophilic lysozyme protein^48^. The IPPase presented much faster relaxation dynamics at 100 MPa and at all temperatures between 298 and 363 K and thus conserved its conformational flexibility in contrast to what was observed at ambient pressure.

## Discussion

**Figure 6:**
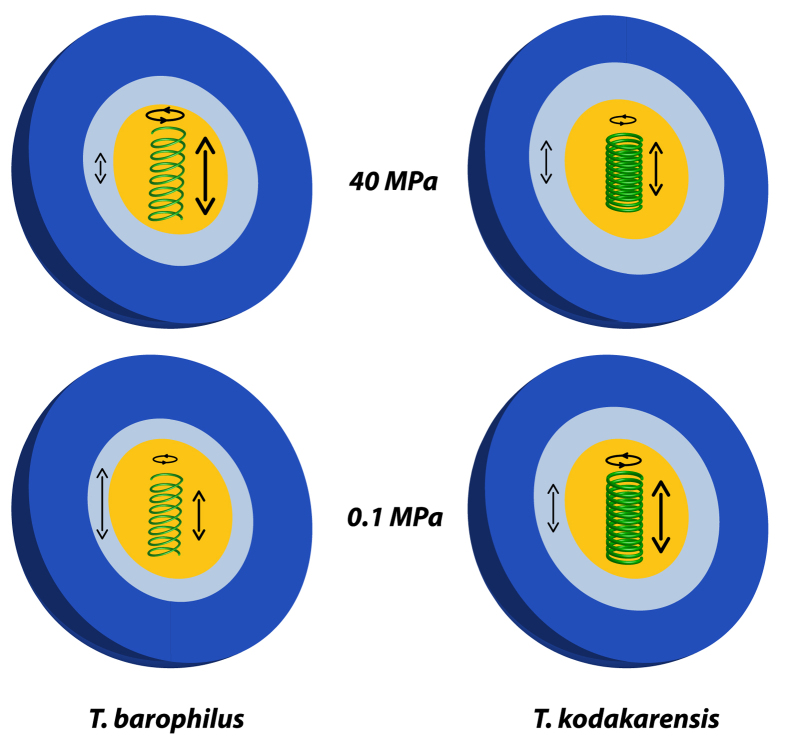
Sketch summarizing the effects of HHP on the two investigated systems. The dark blue surface represents bulk water, the light blue surface hydration water. The green spring characterizes the proteome and its contributions (translation and rotation) to dynamics. The arrow in the light blue area indicates the dependence of the hydration water dynamics on the system and on pressure.

In a previous study on the pressure response of these two organisms, we used elastic incoherent neutron scattering on IN13 over a wide Q-range to demonstrate that *Thermoccocales* had a specific response to pressure changes^41^ characterized by dynamical changes and structural re-arrangements at low pressure and up to a pressure close to the optimal growth conditions. Here we used QENS data, which allows to characterize specific molecular motions, to shed light on the biophysical origin of the pressure adaptation of the piezophilic microorganism, *T. barophilus*. To summarize our findings (cf. Fig. 6), we show that (a) *T. kodakarensis* has a reduced flexibility compared to *T. barophilus* indicating stronger intramolecular non-covalent and hydrophobic bounds; (b) consequently *T. barophilus* has a higher response to pressure application than *T. kodakarensis*; (c) The parameters p_i_ representing the different populations are not very sensitive to pressure application, as they stand only for the number of neutron scatterers present in the sample; (d) The bulk water diffusion coefficients do not depend on the species or on the pressure, which is in agreement with results from other studies in the pressure range investigated here^49^, but the quantity of bulk water is higher in *T. barophilus* than in *T. kodakarensis*; (e) The residence time τ of bulk water molecules increases in *T. barophilus* under pressure application giving a hint for a stronger confinement effect in this state^14^; (f) Concerning hydration water, only a small reduction of the diffusion coefficient was found for *T. kodakarensis* under pressure, whereas *T. barophilus* presents a significant reduction; (g) The proteome has the most surprising behavior: the HWHM Γ_proteome_ is much smaller for *T. kodakarensis* than for *T. barophilus*, confirming the higher stability of the former one. Γ_proteome_ also decreases under pressure for *T. kodakarensis* while it increases for *T. barophilus* indicating an enhancement of the dynamics in *T. barophilus* under HHP.

The comparison of our results with studies on other live cells reported in the literature yield rather similar and encouraging similarities. Indeed, concerning the proportions of the different dynamical populations, QENS experiments on red blood cells have shown a proportion of hydration water of 0.1^10^, and recent THz spectroscopy experiments revealed a proportion of 0.237 of hydration water in HeLa cells^50^, but the authors of the latter study hypothesized that this high content with respect to bacteria or red blood cells was due to the presence of extra organelles in HeLa cells. Therefore, the overall agreement with other studies seems satisfactory. It has furthermore been shown for various biological systems such as *H. marismortui*^9^, *E. coli*^7^, red blood cells^10^ or neural tissue^45^ that the major fraction of water in cells and bacteria (about 80–90%) shows bulk like dynamics with diffusion coefficients in the range of 1–4 10^−5^ cm^2^/s depending on the temperature, what is in compliance with our results. The rotational correlation time, defined as 1/(2 D_R_), with a value of 4.7 ps, is doubled in both samples when compared to typical values found for bacteria^9^, red blood cells^10^ or neural tissue^45^. A hypothesis explaining this increase could be that these two organisms are thermophilic, but the fact that this parameter is unchanged with pressure suggests that it has no role in pressure adaptation.

The dynamical mechanisms of adaptation to HHP seem therefore complex and an interplay between several effects. An overall matter of fact is the higher flexibility of the piezophilic *T. barophilus*, at least up to the pressure values corresponding to the optimum growth conditions. The interaction with water molecules seems to have also a crucial role as the residence time τ of bulk water is changed at 40 MPa, but only for the *T. barophilus* sample. Both calculated values for τ_bulk_ of *T. kodakarensis* and *T. barophilus* at 0.1 MPa are close to that of pure water^44^ (1.1 ps). The higher value observed under high hydrostatic pressure for *T. barophilus* could support a stronger confinement effect. Furthermore, the translational diffusional coefficient of the hydration water D_Thyd_ for *T. barophilu*s is decreased by 35% at 40 MPa, whereas it remains unchanged for *T. kodakarensis*. Since τ_bulk_ most probably reflects the interaction between bulk water and hydration layer, it indicates that even the characteristics of water can slightly change under high pressure application on a living cell due to an increased crowding. These properties are quite peculiar, as experiments on water in presence or absence of proteins^49^,^51^ show that there should be no difference in the diffusion coefficients D of water in the temperature/pressure range explored in the present study. Our results clearly suggest that the specific adaptation to high pressure in *T. barophilus* is done via the modification of the hydration layer properties combined with an increased protein flexibility.

Such findings might seem contradictory, but we should notice that there exists a longstanding debate in literature about a possible master-slave-relationship between surrounding water and biological systems, e.g. it was believed that atoms at the surface of the biomolecule follow the motions of the environment. On one hand, it has indeed been shown that hydration water influences significantly the protein dynamics, although it is not a strong master-slave relationship^52^. Inversely, it was reported that some molecules or solutes can break the structure of the hydrogen-bonded network of neighboring water molecules and thus have a negative hydration effect making the water molecules hypermobile as compared to those in the bulk phase^53^, what illustrates the influence the biological system and hydration water can have on each other. On the other hand, the individual effects on hydration water and on the cells can be understood as follows: 1) hydration water dynamics is reduced due to HHP and Le Chatelier’s principle, 2) whereas the proteome has enhanced dynamics, because it is essential for its correct functioning under high pressure conditions. Indeed, it was shown that flexibility and functionality of proteins are strongly coupled^2^. The “corresponding state principle” was first formulated by Vihinen^54^ and Jaenicke^55^, stating that the flexibility of a protein adapted to extreme conditions should be the same as the flexibility of a protein used to normal conditions. Here it is quantitatively not exactly fulfilled, but the tendency perfectly confirms such principle. Thus, we believe that this mechanism is key to high pressure adaptation.

Besides a specific adaptation mechanism of *T. barophilus* involving a concerted modification of the hydration layer and proteins, we can note that the two organisms differ greatly in the dynamical properties of their proteome. We observe that in the regime measured here, *T. barophilus* has faster protein dynamics than *T. kodakarensis*. The observation that the proteome of *T. barophilus* has a higher flexibility under low pressure conditions was quite unexpected, since this organism requires 40 MPa for optimal growth. In most systems, proteins see a reduction in their dynamics when subject to high pressure conditions, even if this effect is much reduced in crowded solutions like cytosols^56^. The response of the proteome to pressure in *T. barophilus* is thus specific of the piezophilic organism, as was suggested by our previous work using elastic incoherent neutron scattering^41^. The high flexibility of the proteome at 0.1 MPa suggests that it might impact significantly on the proteome function. Consistent with this hypothesis, *T. barophilus* cells were shown to express stress proteins at ambient pressure^57^. This view is further supported by the piezophysiology study of Cario *et al*.^58^ which clearly shows that under low pressure conditions, *T. barophilus* cells accumulate the small organic osmolyte, mannosyl-glycerate (MG). Since the role of osmolytes in cells is to help maintain the proteome in a functional state under stress conditions, the study of Cario *et al*. consequently shows that low pressure is perceived as a stress in this organism. In our experimental conditions, *T. barophilus* cells were grown at 0.1 MPa, and thus accumulated MG to significant levels. We can rule out variations of the cellular concentration of osmolytes during the course of the analysis, since at the temperature of data collection (20 °C), the cells are in a metabolically inactive state. Our results suggest that *T. barophilus* could produce these osmolytes to slow down hydration water and to reduce the hydration layer in order to maintain the biomolecules functional. It is known that organic osmolytes, by modifying the properties of the hydration water can stabilize proteins under different types of stress, including high pressure^51^. Indeed, MG has been shown to increase the rigidity of proteins under thermal stress^59^. Furthermore, the accumulation of osmolytes as a function of HHP has been shown and proposed to constitute part of the adaptation in several species of fish to the increase of pressure with depth in the ocean^60^. Piezosensitive organisms such as the bacterium *E. coli* and the microeucaryote *Saccharomyces cerevisiae*, have pressure tolerant proteome, and express a response under high pressure stress, which clearly indicates a negative impact of increasing hydrostatic pressure on the proteome. In the example of *T. barophilus*, osmolytes are not accumulated under HHP, conditions under which protein dynamics are within the range observed for other biomolecules. The fact that piezophiles have low pressure sensitive proteome is counterintuitive, but it is well supported by evidence obtained by three different approaches as described in this study and^41^,^58^.

Our findings support the fact that it is the interaction with biomolecules who is responsible for the slowing down of the hydration water dynamics. Moreover, other studies have proven that there is a tight dynamical coupling between hydration water and biomolecules^24^,^32^. Our results confirm such effects, and show that this coupling is also present *in vivo*. The increase of Γ_proteome_ under pressure for *T. barophilus* might even point to its capacity to pressure adaptation through a better adapted flexibility in contrast to other biomolecules^61^,^62^,^63^, for which pressure tends to reduce dynamics. Thus, this extreme high flexibility may be the key to high pressure adaptation in this archaeon. For the first time, we are able to propose a scenario for high pressure adaptation in piezophiles at the molecular level.

## Methods

### Sample preparation

The cells of *T. barophilus* and *T. kodakarensis* were cultivated in 3 liters of TRM medium^64^ under anaerobic conditions, at atmospheric pressure and 85 °C until late exponential growth phase (~5 × 10^7^ cells/ml) at the LM2E laboratory in Brest (France). Cells were washed once in isotonic solution and pelleted in a high pressure aluminum capsule under anaerobic conditions. The capsule was inserted in a sealed plastic tube under nitrogen atmosphere, frozen at −80 °C and transferred in dry ice to the ILL, in order to avoid damages due to oxygen contamination during transport.

For the neutron experiments, the thin aluminum cartridges of a diameter of 2 mm and a height of 3 cm were loaded into the high pressure container^41^,^65^ under anaerobic conditions to limit the contact of the samples with oxygen. The sample volume (6 mm diameter) was further filled with D_2_O as a pressure transmission medium which allows to damp the signal from the bulk water (which would hide the others otherwise) and to enhance the signal from the cells and the H_2_O molecules inside the cells or bound to their surface. Under the experimental conditions, the samples were stable for several days at atmospheric pressure. After the neutron experiments, the processed aluminum cartridges were extracted from the high pressure container and shipped back to the LM2E laboratory in Brest for viability tests. Due to the high pressure cell configuration, it was not possible to avoid the exposure of the sample to atmospheric oxygen at opening, during extraction and transport. Since the exposure to oxygen might significantly affect cell survival independently to high pressure and neutron irradiation, in order to have an estimation of cell damage during neutron experiment, we also recorded QENS spectra of the samples at atmospheric pressure immediately after pressure release, and prior to the opening of the high pressure cell (see ESI, Fig. S1).

### Instrumental characteristics

The measurements were performed on the time of flight neutron spectrometer IN5^38^ at the ILL. The incoming wavelength was set to 10 Å, giving an instrumental energy resolution of 10 μeV and a momentum transfer range of about 0.2 < Q < 1.1 Å^−1^. They correspond to a space-time window of up to 32 Å and 100 ps, as can be calculated through Heisenberg’s uncertainty principles. The motions which can be sampled by such a configuration correspond to internal local movements. As HHP experiments request long acquisition times due to the absorption of the cell (typically several hours per point), we had to carefully chose the ranges to be exploited. Moreover, the high-tensile aluminum alloy of the cell does not withstand temperatures higher than 50 °C. Therefore, we selected room temperature in order to get better statistics than at higher temperature and the pressure domain was chosen as to cover the two points corresponding to native conditions of *T. kodakarensis* and *T. barophilus*, e.g. 0.1 and 40 MPa. An extra point was also measured at ambient pressure after pressure release in order to exclude any irreversible processes.

To illustrate the coherence of the results, two figures are added in the ESI of data taken on the spectrometers IN6^39^ (Fig. S2) and IN16B^40^ (Fig. S3) at the ILL. Having different energy resolutions, one being above that of IN5 and the other being below, they reproduce only one aspect of the points discussed here respectively, but they comfort clearly the results.

### Data analysis

Based on the concept of Brownian motion in a fluid it is possible to establish a theory describing diffusion in such a medium. However, fluids are not always formed by identical particles and might contain different dynamical populations (e.g. biomolecules, water molecules and other co-solutes) with individual characteristics. Transposed to the space of transferred energies (ħω) and momenta (with modulus ħQ), to which neutron scattering intensity gives directly access via the structure factor S(Q, ω), the function describing a singular diffusional motion is a Lorentzian curve. The experimental curve of the neutrons’ intensity scattered on various types of particles can thus be fitted by a sum of Lorentzian functions. In theory one could find up to an infinity of Lorentzian curves (cf. equation (1)). In practice the data mostly do not permit to distinguish more than three or four contributions. However, these individual curves characterize specific motions inside the samples and allow extracting typical parameters which depict the motional behavior of one given class of particles. Biomolecules are not fluids and it might be questionable if theories developed for liquids and gases can apply for them. Indeed, motions within proteins, membranes or cells are mainly confined and the atoms have a very restricted space within which they can move freely. However, the HWHM of the Lorentzian functions describing diffusion are often analyzed in terms of models which take into account such confinement in space, as for instance the ‘diffusion in a sphere’ model proposed by Volino and Dianoux^66^ which accounts well for the motions of amino acid side-chains performing diffusive jumps in a spherical volume restricted by neighboring amino acid side-chains. We are analyzing the QENS data according to such an approach in a first approximation to get insights into motions of water molecules and of Hydrogen atoms belonging to the bio-sample.

QENS is the part of scattering where small amounts of energy are exchanged between the neutrons and the sample, giving rise to a broadening of the elastic peak. The structure factor S(Q, ω) describes the scattering signal^28^, which is a function of the modulus of the momentum transfer Q and of the energy transfer Q, both in units of ħ:


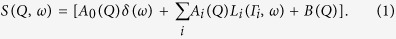


It contains an elastic part, proportional to a delta-function in ω and representing the particles whose motions are not resolved within the instrumental setup, and a sum over Lorentzians *L*_*i*_ which describe each a different motional contribution included in the QENS part. B(Q) accounts for a constant background. For data analysis the structure factor has to be convoluted with the instrumental energy resolution *R*, which can be mimicked by e.g. a vanadium measurement:


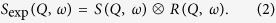


Application of eq. (1) to fit the data is called the “model-free” procedure, which requests many independent fit parameters and there is no known method to determine exactly how much the sum must be extended. If the composition of the sample is approximately known and as typical motions of some molecules present in biological systems, e.g. water, are well characterized, it is possible to use directly a model, which takes such information into account, especially through an analytical expression of the HWHM Γ of the corresponding Lorentzians.

Among the fit parameters which can be determined in such a way are diffusion coefficients D, which specify the (rotational or translational) transport of particles due to a random motion within a limited or an unlimited space and a residence time τ characterizing the typical time a particle performs oscillatory motions before diffusing continuously (Table 1). Some particles can undergo the two corresponding motions simultaneously (oscillations and diffusion) what is mathematically described by a convolution of the two curves. The fit of the experimental data by various contributions permits therefore to decipher several dynamical populations within the samples. In the case studied here, the spectra as function of the modulus of the momentum transferred between neutron and sample, *Q*, could be fitted with a model describing the dynamics of bulk and hydration water, as well as a fast component describing the localized dynamics within the biological system (Fig. 3). We understand by bulk water the water population which has nearly the same characteristics as free water and by hydration water the water molecules bound to the surface (inner or outer) of biomolecules.

As the proportions of water and biomolecules in bacterial cell pellets, with around 70–80% of total water content^43^, are well documented, it was an obvious choice to apply a model that was previously used to analyze QENS data on neurological tissue^45^ which has a similar molecular composition. A very similar approach has furthermore been applied to describe the behavior of multi-lamellar vesicles^67^, membranes^68^ and bacterial cells^26^. It is therefore a recognized approach for complex biological samples. It consists of four different components and a background, convolved with the instrumental resolution function:


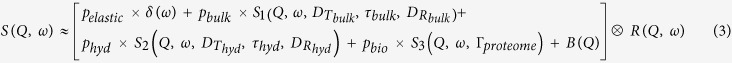


where *p*_*elastic*_ is the elastic fraction describing protons that perform only atomic vibrations (Debye-Waller-like). *S*_*1*_(*Q*, ω) and *S*_*2*_(*Q*, ω) are the scattering intensities arising from the contributions of free water, which has properties very similar to bulk water, and of water molecules bound to the inner or outer surface of the cell. These two components are characterized by a diffusion constant *D*_*T*_, a rotational diffusion constant *D*_*R*_ and a residence time *τ*^67^, which describes the typical time a water molecule performs oscillatory motions around its equilibrium position before diffusing continuously.

An additional contribution, *S*_*3*_(*Q*, ω, G), is needed to optimally reproduce the data. It is related to a faster relaxation found to be described by a large Lorentzian with a constant HWHM Γ_proteome_ as a function of Q and associated with all possible motions within the biological system itself. *p*_*bulk*_, *p*_*hyd*_, *p*_*proteome*_ (with *p*_*elastic*_ + *p*_*bulk*_ + *p*_*hyd*_ + *p*_*proteome*_ = 1) are, respectively, the fractions of atoms corresponding to the various components. These fractions permit to adjust the absolute height of the scattering intensities *S*_*1*_(*Q*, ω)*, S*_*2*_(*Q*, ω) and *S*_*3*_(*Q*, ω) in eq. (3). The described procedure allows discriminating between contributions coming from the cells themselves and from the surrounding water, should it be bound and restricted in its motions or mainly free. The fits were performed by using the program mQfit described in ref. 69, including only the one instrumental resolution of IN5.

The Debye-Waller attenuation factor, a global factor for each QENS curve at constant Q, could be neglected here as we normalized each spectrum. Indeed, it does not introduce additional information for QENS analysis as we were only interested in the line shapes and relative proportions, we had to follow this procedure in order to avoid any problems due to slight miscalculations in the attenuation coefficients.

## Additional Information

**How to cite this article**: Martinez, N. *et al*. High protein flexibility and reduced hydration water dynamics are key pressure adaptive strategies in prokaryotes. *Sci. Rep.* **6**, 32816; doi: 10.1038/srep32816 (2016).

## Supplementary Material

Supplementary Information

## Figures and Tables

**Figure 1:**
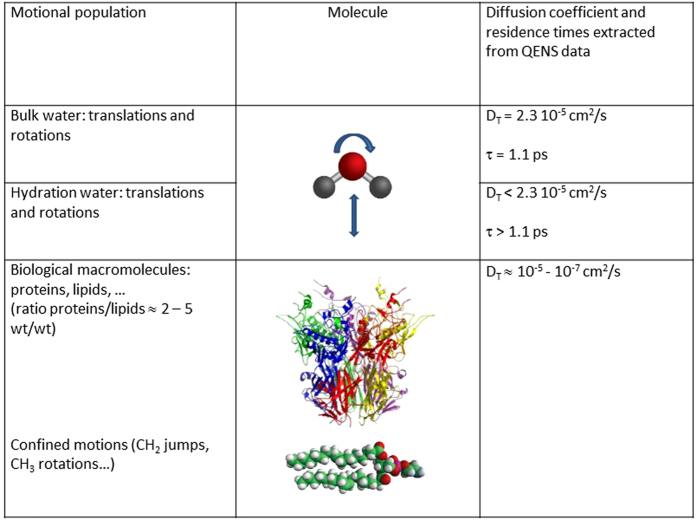
Sketch of the different motional contributions to molecular dynamics measured by quasi-elastic neutron scattering (QENS) in live cells.

**Table 1:** Fit parameter values for the two samples at 0.1 MPa and at 40 MPa.

	*T. barophilus*		*T. kodakarensis*	
	0.1 MPa	40 MPa	0.1 MPa	40 MPa
p_elastic_	0.01 ± 0.01	0.01 ± 0.01	0.03 ± 0.01	0.03 ± 0.01
p_hyd_	0.10 ± 0.01	0.10 ± 0.01	0.17 ± 0.01	0.18 ± 0.02
p_bulk_	0.67 ± 0.04	0.66 ± 0.03	0.60 ± 0.03	0.60 ± 0.03
p_proteome_	0.23 ± 0.03	0.23 ± 0.03	0.23 ± 0.03	0.22 ± 0.03
D_Tbulk_ (10^−5^ cm^2^ . s^−1^)	1.98 ± 0.02	1.98 ± 0.02	1.98 ± 0.02	1.98 ± 0.02
τ_bulk_ (ps)	1.05 ± 0.11	1.55 ± 0.16	1.28 ± 0.13	1.15 ± 0.12
D_Rbulk_ (ps^−1^)	0.11 ± 0.01	0.09 ± 0.01	0.11 ± 0.01	0.11 ± 0.01
D_Thyd_ (10^−7^ cm^2^ . s^−1^)	5.17 ± 0.12	3.34 ± 0.07	4.86 ± 0.10	4.41 ± 0.09
D_Rhyd_ (ps^−1^)	0.06 ± 0.02	0.09 ± 0.03	0.05 ± 0.01	0.05 ± 0.01
Γ_proteome_ (meV)	0.431 ± 0.004	0.451 ± 0.003	0.363 ± 0.005	0.347 ± 0.005
