# Supplementary material for: High protein flexibility and reduced hydration water dynamics are key pressure adaptive strategies in prokaryotes
Source: Sci Rep. 2016 Sep 6;6:32816. doi: 10.1038/srep32816 (PMC5011708; doi:10.1038/srep32816)
Supplement: Supplementary Information [file srep32816-s1.doc]

**Electronic supplemental information**

High protein flexibility and reduced hydration water dynamics are key pressure adaptive strategies in prokaryotes

N. Martinez, G. Michoud, A. Cario, J. Ollivier, B. Franzetti, M. Jebbar, P. Oger, J. Peters

**Integrity of the cells**

We have investigated the overall and mean behavior of cellular components in whole cell pellets of the piezophilic and piezosensitive archaea *T. barophilus* and *T. kodakarensis* as a function of hydrostatic pressure and temperature. These two species are obligate anaerobes and extremely sensitive to oxygen, which leads to cell lysis within minutes. Such sensitivity is further increased in cell pellets, since the amount of reduced water, which acts as a buffering agent towards oxygen, is low. In order to limit the contact between oxygen and the samples at the preparation steps, cells were centrifuged in cylindrical sample holders under N_2_ atmosphere, and the sample holder was frozen at -80°C. Next, the sample holder was inserted in this state in the high pressure cell in a glove box under N_2_ atmosphere. This protocol allowed to avoid exposing the sample to oxygen before the irradiation. In contrast, due to the experimental setup, it was very delicate to avoid contact with oxygen after the neutron experiment, which rendered direct viability tests usually used to determine cell resistance to analysis less pertinent. Such tests were nonetheless performed using the LIVE/DEAD® Cell Viability Assays. They showed that in absence of contact with oxygen, cellular viability remained high (90-95 %), while it rapidly decreased to less than 50% upon exposure to oxic conditions at room temperature. Thus, to evaluate the impact of neutron irradiation and pressure treatment on cell survival, we registered a QENS curve directly after loading the sample into the high pressure cell and one in the same pressure/temperature conditions at the end of the experiment just after the release of pressure. This acquisition showed that neutron had little impact on cells whatever the instrument and time of exposure to neutrons. An example for two such curves representing the neutron intensities summed over the whole accessible range of scattering angles and as function of energy for *T. kodakarensis* is given in figure S1. It demonstrates that pressure application was fully reversible and no deterioration due to neutron radiation or the duration of the experiment could be detected.

**

**

**Figure S1:** Normalized spectra averaged over the scattering angles for *T. kodakarensis*, immediately after loading and after pressure release at the end of the experiment.

**QENS acquisition on IN6 and IN16B**

We investigated samples of the same two organisms under the same pressure and temperature conditions on the spectrometers IN6^39^ (Fig. S2) and IN16B^40^ (Fig. S3) at the ILL. These instruments have energy resolutions different from IN5. IN6 was used in a configuration with ΔE ≈ 80 μeV, corresponding to a time window Δt ≈ 10 ps, and IN16B has a resolution ΔE ≈ 1 μeV, corresponding to a time window Δt ≈ 1 ns. Therefore, on IN6 one probes essentially bulk water dynamics and the hydration water and proteome dynamics are hidden in the background, whereas one probes proteome and hydration water dynamics on IN16B.

Fig. S2 reproduces well that *T. Kodakarensis* is stiffer than *T. barophilus*, but pressure does not seem to influence the dynamics of the bulk water, what reinforces our findings on IN5.


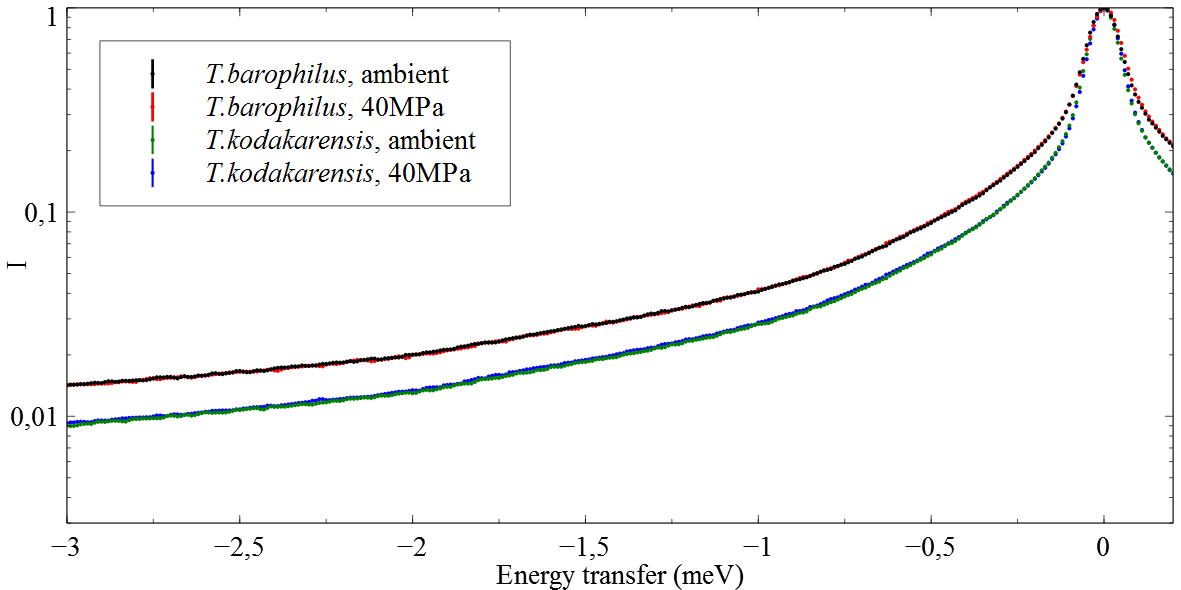


**Figure S2:** Normalized spectra averaged over the scattering angles for both samples measured on IN6 at the ILL.

On IN16B, we summed the elastic intensities over all scattering angles and normalized them to ambient pressure. It permitted to well reproduce the high pressure effect on the hydration water and proteome dynamics of *T. barophilus*, whereas it is almost unaffected for *T. kodakarensis*.

**

**

**Figure S3:** Elastic intensities summed over the scattering angles for both samples measured on IN16B at the ILL.
